# Differences in the limb blood flow between two types of blood flow restriction cuffs: A pilot study

**DOI:** 10.3389/fphys.2022.931270

**Published:** 2022-07-26

**Authors:** Tom Citherlet, Sarah J. Willis, Audrey Chaperon, Grégoire P. Millet

**Affiliations:** ^1^ Institute of Sport Sciences, Synathlon, University of Lausanne, Lausanne, Switzerland; ^2^ Department of Biological Sciences, University of Denver, Denver, CO, United States

**Keywords:** vascular occlusion, BFR, BStrong, Hokanson, ultrasound

## Abstract

**Introduction:** The determination of the optimal occlusion level is a key parameter in blood flow restriction (BFR). This study aimed to compare the effects of elastic (BStrong) vs. nylon (Hokanson) BFR cuffs on blood flow in the lower and upper limbs.

**Methods:** Eleven healthy participants undertook several BFR sessions with 2 different cuffs of similar width on their lower and upper limbs at different pressures [200, 250, 300, 350, and 400 mmHg for BStrong and 0, 40, and 60% of the arterial occlusion pressure (AOP) for Hokanson]. Doppler ultrasound recorded blood flows through the brachial and femoral artery at rest.

**Results:** With BStrong, only 350 and 400 mmHg pressures were significantly different from resting values (0% AOP). With Hokanson, both 40% and 60% of the AOP were significantly different from resting values (*p* < 0.05).

**Discussion:** While both cuffs elicited BFR, they failed to accurately modulate blood flow. Hokanson is appropriate for research settings while BStrong appears to be a convenient tool for practitioners due to its safety (i.e., the impossibility of completely occluding arteries) and the possibility of exercising freely detached from the pump.

## Introduction

Blood flow restriction (BFR) is a training method that attracted scientific interest very early (Shinohara et al., 1997). It has been since increasing in popularity. This method consists of restricting blood flow via occlusion cuffs placed proximally on limbs during training exercises, for example, weightlifting. BFR partially restricts arterial inflow and generally, fully restricts venous outflow. Numerous studies to date have shown beneficial muscle adaptations of the BFR method. There is a general consensus that low-load BFR training induces gains superior to low-load training, but does not surpass that of traditional high-load resistance training (Hughes et al., 2017; Patterson et al., 2019). The mechanisms responsible for increased strength and muscle gains appear to be multicausal. It has been suggested to be related to metabolite accumulation, fast-twitch fiber recruitment, and mTOR signaling (Wernbom et al., 2008; Loenneke et al., 2010; Scott et al., 2014).

The use of BFR has been shown to be beneficial for populations such as athletes, the elderly, or patients undergoing rehabilitation. Importantly, it has been thus far approved as a safe method for healthy subjects (Loenneke et al., 2011). It has also been applied to at-risk populations (e.g., with obesity, diabetes, cerebrovascular diseases, neuromuscular diseases, orthopedic diseases, respiratory diseases, hypertension, or cardiac diseases) (Nakajima et al., 2006).

Applied pressure plays an important role in BFR. When pressure is too low, no blood flow, metabolic, or muscle changes are observed while a too high pressure may increase discomfort without further blood flow decrease (Mattocks et al., 2017). It has been recommended to set applied pressure based on the measurement of arterial occlusion pressure (AOP) since it takes into account the characteristics of the cuff and the individual, which account for large influences in the occlusion stimulus (Patterson et al., 2019). For example, larger limb sizes require greater pressure (Loenneke et al., 2012; Barnett et al., 2016) and wider cuffs require lower pressure (Mattocks et al., 2018; Patterson et al., 2019). However, it is still unclear if cuffs of the same size, but different materials present significant differences. This latter point is of primary importance considering the wide variety of cuffs available on the market. In the literature, elastic or nylon cuffs have been mostly used. Nylon and elastic cuffs of the same size (5 cm) have been compared but no significant difference was found in the AOP measured at rest in the supine position in the lower body (Loenneke et al., 2013). In another study, the authors chose to measure repetitions to exhaustion as a surrogate marker of blood flow changes. They found no difference in repetition to exhaustion and perceptual responses between narrow elastic and narrow nylon cuffs of the same width (Loenneke et al., 2014). Conversely, it was shown that elastic cuffs had higher AOP than nylon cuffs of similar width at rest in the upper body (Buckner et al., 2017). That said, when applied at the same %AOP, there were no differences in the repetitions to volitional failure suggesting that the reduction in blood flow was similar between cuffs. Thus, differences in cuff material could be corrected simply by using the % AOP (Patterson et al., 2019). In addition, novel BFR equipment cannot occlude completely blood flow (e.g., BStrong) and for this reason prevents the risks associated with the potential unsafe use of the cuffs (e.g., excessive duration and level of the pressure applied). However, the %AOP cannot be used with this characteristic.

Therefore, given the controversial results regarding the material effect on AOP, this study aimed to compare a new and less costly elastic cuff system (BStrong) to a rigid nylon cuff system commonly used in the literature (Hokanson) on blood flow in lower and upper limbs measured at rest in a sitting position. From a practical point of view, it also intended to provide recommendations on the pressures to be applied when using BStrong cuffs. We hypothesized that elastic cuffs would require higher pressure than nylon cuffs to obtain the same BFR.

## Methods

### Experimental approach to the problem

This study evaluated the blood flow with Doppler ultrasound after applying different pressure levels in random order with two cuffs systems (BStrong vs. Hokanson) in the lower and upper limbs.

### Participants

Eleven healthy subjects (7 women and 4 men) agreed to participate in this study. Participants’ age, height, body mass, systolic and diastolic blood pressures were 26.3 ± 3.7 years, 173 ± 8 cm, 69.6 ± 13.7 kg, 123.4 ± 19.3 mmHg, and 75.5 ± 12.7 mmHg, respectively. The participants did not have any injury and no skeletal or muscle pain in the past 3 months. In addition, participants were required to have no blood clotting problems, nor be consuming aspirin or anticoagulants. Furthermore, no participants had performed any intense training before the start of the experiment. The local Ethical Committee approved the entire experimental protocol (2018–02298), and participants gave their written informed consent.

## Material

The Hokanson model 10 cm cuff (SC10, 11 × 85 cm cuff size, 10 × 41 cm bladder size; Hokanson, Bellevue, WA, United States) was used for the lower body and the 5 cm cuff (SC5, 6 × 83 cm cuff size, 5 × 41 cm bladder size; Hokanson, Bellevue, WA, United States) was used for the upper body. To allow for the best possible comparison, BStrong cuffs that most closely resembled the Hokanson system in width were chosen: the yellow cuff (BStrong, Park City, UT; 54–79 cm long; 7.5 cm wide), which is the widest, was chosen for the lower body, and the green cuff (18–31 cm long; 5 cm wide) for the upper body. The red cuff (26–45 cm long; 5 cm wide) was used only on one participant’s upper body, which had a larger arm circumference.

### Procedures

After verifying eligibility, the blood pressure was measured with an automatic blood pressure monitor (Omron RX-I, model HEM-632-E) at rest in a sitting position to identify any potential hypertension. Measurements were taken at the right wrist with the arm at the level of the heart. Two blood pressure measurements were taken and averaged. In addition, the circumference of the limbs was measured on all recorded limbs using metric tape. The measurements with either BStrong or Hokanson started in a randomized order. The first limb and the first side to be occluded were also randomly assigned. A break of 2 min was observed between each measurement. Throughout the measurements, the participants remained seated at rest on a chair with feet flat on the floor.

As reported above, the maximal available pressure with BStrong does not lead to arterial occlusion while lower pressures completely occlude blood flow with Hokanson. Therefore, using the same pressures with the two cuffs models would not have been appropriate and thus, different levels of pressure between both systems were used.

The experiment with BStrong started by placing the deflated elastic cuff on the proximal part of the limb to be occluded. The pressure in the cuff was increased using a hand-held pressure gauge up to 200, 250, 300, 350, and 400 mmHg randomly to account for possible effects of time order. Each time, once the pressure was maintained for 1 min, blood flow was recorded for 30 s by a linear Doppler (L12-5L60N) coated with ultrasound gel placed on either the brachial or femoral artery. Doppler ultrasound was used to obtain a real-time image of the measured artery using EchoWave II 3.4.4 software (version 3.4.4, Telemed Medical Systems, Telemed Ltd. Lithuania, Milano, Italy).

The experiment with Hokanson was conducted similarly except that it started with the determination of the AOP and that the pressure was increased up to 0%, 40%, and 60% of the AOP in random order. To determine AOP, the blood flow was detected with the Doppler probe, and the pressure in the cuff was gradually increased using an E20 Rapid Cuff Inflator (Hokanson, Bellevue, WA, United States) until no blood flow was detected in the artery, which was defined as the AOP. The cuff was deflated immediately afterwards. This measurement was repeated 2 to 3 times and averaged to record an accurate and reliable AOP value.

### Data analysis

The data were processed using EchoWave II software. The diameters of the brachial and femoral arteries were measured manually using digital calipers. The values were averaged over 10 measurements throughout 30 s of recording for each of the 8 pressures applied with the two cuff systems (0%, 40%, and 60% with Hokanson cuffs and 200, 250, 300, 350, and 400 mmHg with BStrong cuffs). Based on the diameter of the vessel (in mm), the EchoWave software allowed calculation of the blood flow values (ml⋅min⁻^1^). The mean ± SD were thereafter calculated for each limb. Outliers, i.e., values of more than ±2 SD from the mean were removed.

### Statistical analysis

Data are presented as mean ± SD. A repeated-measures three-way ANOVA was used to compare resting blood flow values (ml⋅min⁻^1^) between different pressures between the two types of cuffs (BStrong vs. Hokanson) but also to compare whether there was a significant difference between the arms or legs and between the left or right side. The normality of the data distribution was assessed using the Shapiro-Wilk test. Mauchly’s test was used to test the assumption of sphericity. Where this assumption was violated (*p* < 0.05), the Greenhouse-Geisser (if Epsilon <0.75) or Huynh-Feldt (if Epsilon >0.75) corrections were used. In the case of a significant interaction, multiple comparisons (post hoc tests) corrected using the Tukey method were performed. Effect sizes were calculated using Cohen’s methods. The threshold for statistical significance was set to *p* < 0.05. All data were analyzed using Jamovi statistical software version 1.1.9 (Jamovi Project, Sydney, Australia).

## Results

Table 1 presents the measurements of the arms and legs circumference of each participant and the pressures that were needed to obtain a complete arterial occlusion with the Hokanson system. The average AOP was 208.5 ± 29.2 mmHg with Hokanson, whereas such pressure applied using BStrong did not lead to blood flow significantly different from resting values (0% of the AOP). Even the highest BStrong pressure (400 mmHg) did not completely occlude blood flow.

**TABLE 1:** Arterial occlusion pressures (AOP) measured at rest with Hokanson and circumference of participants’ limbs.

Participants	Right arm		Left arm		Right leg		Left leg	
	Circumference (cm)	AOP (mmHg)	Circumference (cm)	AOP (mmHg)	Circumference (cm)	AOP (mmHg)	Circumference (cm)	AOP (mmHg)
1	26	200	25.5	189	54	254	54	200
2	29.4	202	29.1	210	59.6	259	59.2	183
3	32.7	197	32.5	210	60.4	204	60.8	214
4*	29.1	190	29.6	192	58.1	178	58.2	156
5	30.6	227	30.2	186	56.8	206	56.7	198
6*	34.6	222	35.7	257	58.3	223	59.4	231
7	32.2	237	31.4	256	58.2	208	56.7	190
8	36.7	244	N/A	N/A	67.1	262	N/A	N/A
9	34.2	217	N/A	N/A	60.9	217	N/A	N/A
10	N/A	N/A	29.2	216	N/A	N/A	54.6	181
11	N/A	N/A	27.8	160	N/A	N/A	53.8	142
Mean	31.7	215.1	30.7	208.4	59.3	223.4	58.1	188.3
SD	3.3	18.9	3.4	32	3.6	29	4.1	27.4

*Left-handed participants. N/A because only one side was measured.

**FIGURE 1:**
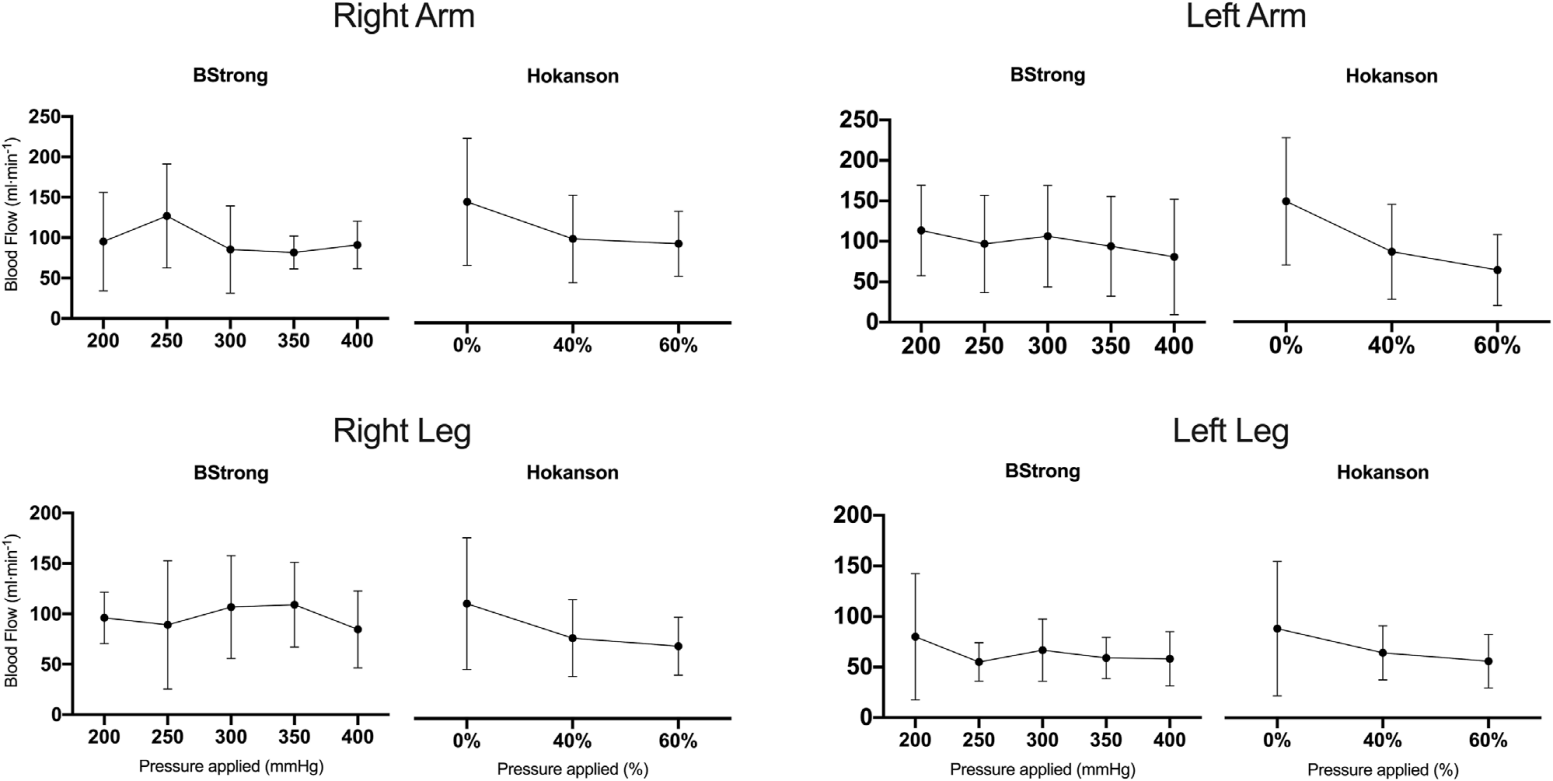
Blood flow values (mean ± standard deviations) of right arm, left arm, right leg, and left leg. For both arms and legs, BStrong cuffs were inflated up at 200, 250, 300, 350, and 400 mmHg while Hokanson cuffs were inflated up to 0% (resting values), 40%, and 60% of arterial occlusion pressure.

Figure 1 represents the blood flow values with BStrong and Hokanson systems in all four limbs. The two BFR systems were able to significantly reduce blood flows compared to resting values. A pooled analysis between limbs revealed that, with BStrong, only 350 (*p* = 0.016, *d* = 0.688) and 400 mmHg (*p* = 0.002, *d* = 0.805) pressures were significantly different from resting values (0% AOP). With Hokanson, both 40% (*p* = 0.009, *d* = 0.715) and 60% (*p* < 0.001, *d* = 0.948) of the AOP were significantly different from resting values (*p* < 0.05). However, while the two highest BStrong pressures decreased the blood flow compared to resting values (0% of the AOP), BStrong was not able to regulate blood flow according to the pressure applied (no significant differences were found between each of the pressure applied). This was also the case with Hokanson, however to a lesser extent. The blood flows tended to slightly decrease as the pressure in the cuff was increased. Significant differences were found between 0 and 40% of the AOP (*p* = 0.009) but not between 40 and 60% of the AOP. In addition, there were significant differences in blood flow between limbs (*p* = 0.017, *d* = 0.487) and sides (*p* = 0.028, *d* = 0.287).

## Discussion

This study aimed to compare two cuff systems (BStrong vs. Hokanson) of different materials (elastic vs. nylon, respectively) with similar widths. This is the first work to compare BStrong to Hokanson, a device commonly used in research. It also strived to describe the blood flows that can be expected with different levels of pressure. The recommendations on the occlusion pressure levels are therefore limited to blood flow acute responses and cannot be generalized since we did not investigate either other important criteria (e.g., discomfort) or prolonged physiological adaptations.

BStrong (with 350 and 400mmHg), as well as Hokanson (with 40 and 60% of the AOP), were able to significantly decrease blood flow compared to resting values. However, BStrong, and Hokanson to a lesser extent, struggled to modulate blood flow according to the pressure applied.

BStrong did not significantly decrease blood flow with increasing pressures but showed rather variable blood flows with increasing pressures (Figure 1). Specifically, no significant differences were observed in blood flow between each measured pressure (200, 250, 300, 350, and 400 mmHg). Only the blood flow values with BStrong inflated to a pressure of 350 or 400 mmHg were significantly different from the values at rest (0% AOP). It has been recommended to set BFR pressure as a percentage of the AOP, but it is not possible to do so with BStrong due to its pressure range limit. In regard to blood flow being a targeted variable, the presents results suggest that a pressure equal to or superior to 350 mmHg should be applied in the lower and upper limbs with BStrong.

Hokanson was able to decrease blood flow with increasing levels of pressure, however this was not a linear relationship (Figure 1). Blood flow was significantly decreased between 0 % and 40% of the AOP but not between 40 and 60% of the AOP. Consistent with the results of other studies, no significant difference between pressures ranging from 40 to 80% of the AOP on the legs at rest (Crossley et al., 2020) and between 40 and 60% of the AOP on the upper body (Mouser et al., 2017, 2018) were reported. The current results suggest that pressures as low as 40% of the AOP may offer a comparable restrictive stimulus to higher ones but at more comfortable pressures. This suggestion is reinforced by a study that demonstrated that 8 weeks of BFR training with 40 % vs. 90% of the AOP had similar effects on muscle size and function (Counts et al., 2016).

The profiles of the blood flow changes suggest that Hokanson is a better option for clinical practice while, on the other hand, BStrong seems to be applicable in the practical setting, due to its removable pump and its lower risks associated with the potential wrong use of the cuffs since it is not possible to completely occlude the arteries with this cuff system. These results also highlight that more pressure doesn’t always mean less blood flow and that there is variability in the blood flow obtained with BFR. This variability should be considered when practicing BFR. It is hypothesized that several factors may have contributed to this blood flow variability such human error in blood flow measurement, variability in the restrictive stimulus due to the BFR system, duration of occlusion during measurements or duration of rest in-between the measurements.

### Cuffs comparison: Individualization, safety, and material

Although it is recommended in the literature to use custom pressures based on a relative percentage of AOP, it cannot be done with BStrong because its pressures range limits the possibility to fully occlude the arteries. Conversely, Hokanson occluded blood flow with its automatic inflation system with an average of 208.5 ± 29.2 (Table 1 for limb by limb averaged pressures). This important difference renders the individualization of the pressures more difficult/not possible with the BStrong cuff system but reduces the risk to its users and thus improves the safety of its use for the public.

The present results showed a significantly higher average blood flow during restriction in the arms than in the legs (*p* = 0.017) (Figure 1). This could be due to different hemodynamic regulatory mechanisms in response to BFR in the arms vs. the legs. Indeed, another study observed greater deoxygenation and greater blood volume changes in arms vs. legs during repeated sprint tests in hypoxia (Willis et al., 2019). This would suggest greater vascular reactivity of the arms than legs and could explain the present results.

There was a significant difference in blood flows between the left and right sides (*p* = 0.028). Although not significant, the larger limb’s circumferences on the right side (Table 1) could explain this variability (Figure 1). Indeed, the larger the limb, the more pressure in the cuff is needed to reduce blood flow (Loenneke et al., 2012; Jessee et al., 2016). Likewise, when comparing the AOP values obtained in the limbs (Table 1), higher pressure was required to occlude the limbs on the right side than those on the left side. This would highlight the importance of the recommendation that pressure should be established based on the circumference of the limb to ensure safety (Jessee et al., 2016) but also provide a similar stimulus for all participants (Mouser et al., 2018).

The material could likely explain why only 208.5 ± 29.2 mmHg in average leaded to arterial occlusion with Hokanson while higher pressures did not lead to significant difference from resting values (0% of the AOP) with BStrong. Indeed, due to the more rigid nylon character, Hokanson cuffs are stretched less easily than elastic cuffs and therefore compress more soft tissues. Therefore, cuffs constructed of more rigid materials would occlude the limbs with less pressure than more elastic cuffs. Similarly, Buckner et al. (2017) found resting AOP higher with nylon cuffs (Hokanson, Bellevue, WA, United States) than with elastic cuffs (Kaatsu Master, Tokyo, Japan) of the same width in the upper body while Loenneke et al. (2013) observed no significant difference between elastic (Kaatsu Master, Tokyo, Japan) vs. nylon (Hokanson, Bellevue, WA, United States) cuffs of similar size in the lower body. These opposite results suggest that there may even be differences between elastic cuffs. It is therefore important to consider the type of cuff used, as well as its width (Mattocks et al., 2018; Patterson et al., 2019) when practicing BFR, because it may lead to a completely different restriction stimulus.

### Limitations

The small sample size of this study (*n* = 11) related to the available resources underlines the need for further investigation. It should also be noted that the measurements were taken while resting in a seated position. The use of this information relative to seated rest must be interpreted with caution when applying to participants during exercise. Exercise, causing increased blood flow to the working muscles, is likely to need higher pressure to restrict comparably arterial flow. Despite these limitations, the present results provide an important adjunct to the BFR literature, by analyzing the differences between two cuff systems of similar size but different materials and pointing up the variability of blood flow restriction.

### Practical applications

These results underline first the variability in blood flow that can occur when applying pressure with different BFR cuffs materials. This variability should be kept in mind when practicing BFR. The users should therefore not expect a linear decrease in blood flow when increasing the pressure.

Current results suggest that a pressure ≥ to 350 mmHg should be applied with BStrong and that pressure as low as 40% of the AOP can be applied to achieve a BFR comparable to higher (60%) and less comfortable pressures with Hokanson. Despite the importance of restrictive pressure application, the effectiveness of BFR training also depends on the protocol chosen. The practitioner must adjust training variables (load, intensity, volume) to achieve muscle changes (Patterson et al., 2019). In addition, it was suggested that the AOP increases with exercise so it should be taken into account when trying to set an optimal relative pressure (Barnett et al., 2016).

BStrong did not decrease blood flow with increasing pressures, it seems therefore not possible to modulate blood flow with BStrong. On the other hand, Hokanson decreased blood flow with increasing pressures, but not significantly, it seems for this reason roughly possible to modulate blood flow with Hokanson. It is also worth mentioning that the recommended BStrong pressure levels in the current study are even superior to the AOP obtained with Hokanson which could increase discomfort. Beyond these results, BStrong has practical considerations (e.g., training and/or rehabilitation) as the pump can be removed from the valve once inflated which allows the users to move freely instead of performing exercise while connected to an inflation system.

## Data Availability

The raw data supporting the conclusions of this article will be made available by the authors, without undue reservation.

## Abbreviations

AOP, Arterial occlusion pressure; BFR, Blood flow restriction; bSBP, Brachial systolic blood pressure.
